# Full Immunization Coverage and Associated Factors among Children Aged 12-23 Months in a Hard-to-Reach Areas of Ethiopia

**DOI:** 10.1155/2019/1924941

**Published:** 2019-05-27

**Authors:** Abadi Girmay, Abel Fekadu Dadi

**Affiliations:** ^1^Tigray Regional State Health Office, Ethiopia; ^2^Department of Epidemiology and Biostatistics, Institute of Public Health, College of Medicine and Health Sciences, University of Gondar, Gondar, Ethiopia

## Abstract

**Introduction:**

Childhood immunization averts 2.5 million annual child deaths globally. However, poor monitoring, possibly due to a lack of locally available data on immunization, might affect full protection of vaccines from Vaccine Preventable Diseases. This study was aimed at bringing data about immunization service coverage and its associated factors from Sekota Zuria district, which is one of the hard-to-reach areas in Amhara Region, Ethiopia.

**Methods:**

A community-based cross-sectional study was conducted from September 20 to October 28, 2017, among 620 children aged 12-23 months in seven randomly selected rural kebeles of Sekota Zuria district. Socio-demographic child conditions and vaccine-related data were collected using a pretested interviewer-administered questionnaire. Multivariable logistic regression analysis was carried out to identify factors associated with immunization coverage at a p-value ≤ 0.05. Crude and Adjusted Odds Ratio (AOR) with their confidence interval were reported.

**Results:**

77.4% (95%CI: 74.0%-80.6%) of children aged 12-23 months were fully immunized. Having antenatal care visit (AOR=2.75, 95%CI: 1.52-5.0), higher level of maternal education (AOR=2.39, 95%CI: 1.06-5.36), mothers' good knowledge on immunization (AOR=3.70, 95%CI: 2.37-5.79), short distance to health facility (AOR=2.65, 95%CI: 1.61-4.36), and being born in health institutions (AOR=2.58, 95%CI: 1.66-3.99) had increased the odds of full immunization coverage while having five and more family size reduced the odds of children's vaccine uptake (AOR=0.62, 95%CI: 0.38-0.99).

**Conclusion:**

Full immunization coverage of the district was lower than the target set by the World Health Organization. Improving mother's health seeking behavior toward pregnancy follow-up and enhancing mothers' knowledge on child immunization, strengthening outreach services, community engagement, and actively working with local community-based health agents are recommended to increase number of children to be vaccinated.

## 1. Introduction

Childhood immunization is one of the most valuable public health interventions available [1] for preventing childhood morbidity and mortality. Basic immunizations are estimated to avert 2.5 million annual child deaths globally from diphtheria, tetanus, pertussis, and measles [2]. Despite this enormous use, immunization coverage in developing countries has reported to being low [3]. In 2011 alone, 1.5 million children died from Vaccine Preventable Diseases (VPDs) [2, 4]. If all currently available vaccines were widely adopted and every country attained at least a 90% vaccine coverage, millions of lives would have been saved globally [4].

In 2014, about 18.7 million children did not receive the 3rd dose of Diphtheria-Pertussis-Tetanus (DPT3) vaccine and 70% of them were live in ten developing countries including Ethiopia [2, 5]. Global Vaccine Action Plan (GVAP) had put a goal to reach immunization coverage of at least 90% in every nation and at least 80% of DPT3 coverage in every district by 2015 [6]; however, it was only achieved by 56 of the 194 WHO member states [5]. The DPT3 coverage has increased from 74% in 2010 to 80% in 2014 in Africa with great disparities among countries. Most countries in Sub-Saharan Africa (SSA) experienced a DPT3 coverage that reached less than 50% [6]. In 2014 measles vaccine coverage in Africa was 74% and as a result estimated 48,000 measles related-deaths were occurred [6].

In Ethiopia, the Expanded Program on Immunization (EPI) program emphasizing six vaccines has been given on a routine and outreach basis since 1980 [7–10]. Ethiopia follows the WHO immunization schedules and provides the following vaccines based on the specified schedules: one dose of Bacillus Calmette-Guerin (BCG) and initial dose of oral polio vaccine (*OPV*_*O*_) given at birth; three doses of each Pentavalent (DPT‐HepB‐Hib), OPV, and Pneumococcal Conjugate Vaccine (PCV) given at 6th, 10^th^, and 14th weeks; two doses of Rotavirus vaccine given at 6th and 10th weeks; and lastly measles vaccine at 9th month [7, 9, 11].

Expanding immunization service is among one of the Ethiopian child survival strategies targeted to protect nearly 3-million annual births against VPDs [12] but a significant portion of children has not been immunized [13]. As a result deaths in the first few years of life in Ethiopia are among the highest in the world and many of these were believed to be attributed to VPDs [8, 14].

Though, full immunization coverage has been raised from 24% in 2011 to 39% in 2016 EDHS report [14, 15]; this achievement remains far below the goal set in the 4th Health Sector Development Plan (HSDP-IV) and the GVAP target plan to achieve 90% coverage nationally and 80% in every district for all vaccines by 2020 [6, 9].

Studies have also reported maternal education, access to health services, family socioeconomic status, child place of delivery, antenatal care (ANC) visits, mothers TT immunization status, knowledge on immunization, sex of the child, place of residence, religious affiliation, and exposure to mass media as predictors of poor immunization coverage [9, 11, 16–18]. However, the relationship of these factors in predicting full immunization has not always been consistent across study areas. Measles and pertussis are frequently reported and outbreaks of these diseases are recurrently occurring in Sekota Zuria District. Therefore, we aimed at determining full immunization coverage and identifying its associated factors in this district.

## 2. Methods 

### 2.1. Study Setting and Design

A community-based cross-sectional study was conducted from September 20 to October 28, 2017, in Sekota Zuria district, Wag-Himra Zone, Amhara Regional State, and Northern Ethiopia. Sekota Zuria is one of the seven districts in Wag-Himra Zone, Amhara Regional State, and found 720kms away from Addis Ababa, the capital of Ethiopia. The district is claimed as one of the hard-to-reach areas in the region and has an estimated area of 1671.56 km^2^ and 33 rural “kebeles” (the lowest administrative unite). The total population of the district based on projections from 2007 census was 138,846 in 2017, of which under five children account for 13.5% (4,319). The district has seven health centers and 33 health posts, which provide a routine vaccination service for their catchment population.

### 2.2. Population and Sampling

Children aged 12-23 months who were living in seven randomly selected kebeles of the district were included while those children whose mothers/caregivers found to be mentally/critically ill during the data collection period were excluded from the study. The required sample size was determined by comparing sample sizes obtained from single and double population proportion formulas. For a single population formula, the following assumptions were considered: the proportion of fully immunized children aged 12-23 months from EDHS 2011 which was 24.3%, 95% confidence level, 5% margin of error, % nonresponse rate, and a design effect of 2. The sample size was calculated to be 623. In the double population proportion formula, factors significantly associated from previous studies such as maternal education, institutional delivery, and mothers' TT immunization were used to calculate the sample sizes using Epi-Info7 StatCalc program using 95% CI, reported odds ratio, and power of 80% [19]. Finally, 623 was found as the biggest sample size of all approaches and considered as a final sample size of the study.

**Figure 1:**
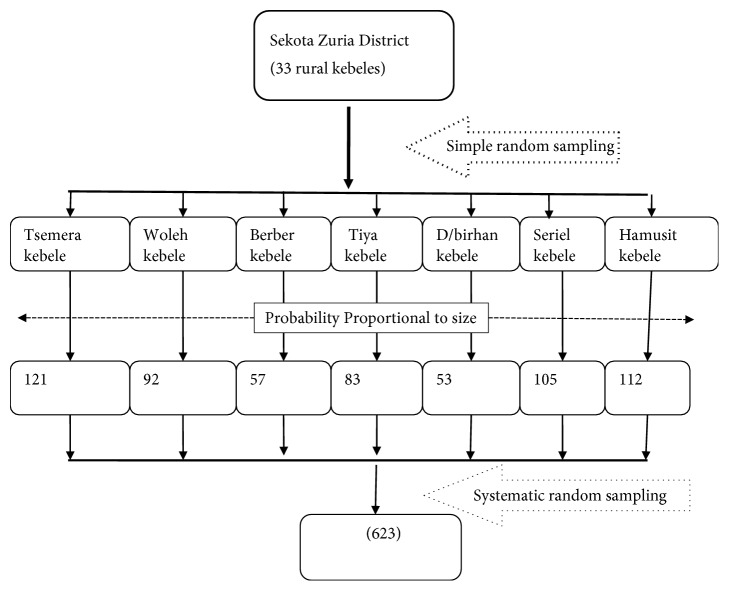
Sampling framework of immunization coverage and associated factors among children aged 12-23 months in Sekota Zuria district, 2017.

Study participants were selected by multistage sampling technique. Stage one, seven kebeles were randomly selected and stage two households with children aged 12-23 months were randomly selected and included in the study. Community Health Information System (CHIS) registration log was used as a sampling frame for the selection of households with children 12-23 months. If respondents were not found at home during the data collection, interviewers have revisited the households for the second time and when the interviewers failed to find the eligible participant after two visits, the next household was contacted. (Figure 1)

### 2.3. Data Collection

The data collection tool was adapted from EDHS 2011 immunization questionnaire and other relevant literature. It was first prepared in English and translated to Amharic language and back-translated to English to check the consistency of translation. The questionnaire mainly included immunization histories of children, socio-demographic characteristics of mothers, maternal health care utilization, and knowledge of mothers on immunization. Information on vaccination coverage was collected in two ways: from the child vaccination card or from mothers' verbal report. Information from the child card was extracted in cases where child immunization card was available. When there was no vaccination card for the child or if a vaccine had not been recorded on the card as being given, the mothers were asked to recall the specific vaccines given to her child. The information obtained from the child card was taken in the case where both conditions have been met.

Six diploma nurse data collectors and two supervisors were recruited in the data collection. Data collectors and supervisors were trained on overall data collection procedures and the techniques of interviewing. Before starting the actual data collection, the questionnaire was pretested on 5% of similar respondents in other kebeles of the district which was not included in the final study. All field staffs and the principal investigator have assessed the clarity and completeness of the questionnaire. The collected data was checked for completeness, consistency, accuracy, and clarity by the supervisors and the principal investigator on a daily basis.

### 2.4. Data Processing and Analysis

Data were cleaned and entered to Epi-Info7 [20] and transferred to SPSS version 20 [21] for further analysis. A child aged 12-23 months who received all currently recommended vaccines (one dose of BCG, three doses of Pentavalent, three doses of OPV (excluding OPV0 which provides at birth), three doses of PCV, two doses of Rotavirus vaccine, and one dose of measles vaccine) any time before the data collection was taken as fully vaccinated. Conversely, a child aged 12–23 months who missed at least one dose of the recommended vaccines or not vaccinated at all was considered as not fully vaccinated. Data were presented using descriptive statistics. We have checked for interaction terms and multicollinearity was also not found. In the bivariable analysis, independent variables significantly associated with the dependent variable at P-value ≤ 0.20 were included in the multivariable logistic regression analysis and variables significantly associated at p-value ≤ 0.05 were identified as predictors of immunization status. Backward stepwise regression method was used to select the variables. The degree of association was assessed using crude and adjusted odds ratios. Adequacy of the model was assessed using Hosmer and Lemeshow goodness of fit (P-value =0.308).

### 2.5. Ethical Issues

Ethical clearance was obtained from Institutional Review Board of University of Gondar. Permission letter was received from Sekota Zuria district health office. Verbal informed consent was obtained from each respondent prior to data collection. Participants were fully informed about the objectives and procedures of the study and their right to refuse participation at any time during the study. Study participants were also informed that all data obtained from them would be kept confidential. At the end of each interview, mothers found to have nonimmunized or partially immunized children were advised to vaccinate their child and to follow the regular immunization sessions.

## 3. Results 

### 3.1. Background Characteristics of the Study Population

Six hundred twenty mothers of children aged 12-23 months were included in the study, which makes a response rate of 99.5%. A mean and standard deviation (±SD) of the age of mothers was 29.3 (±7.2) years. The mean age of the child was 16.7(±3.39) months and 51.0% of them were females. Half (49.9%) of the mothers were not able to read and write, while 11.8% of them have attained a secondary or higher level of education. About 95% of the respondents were Orthodox Christian while 5.0% of them were Muslims. Nearly 41.0% of the households had a family size of four or below and 40.8% of the households had an average monthly income between 500 and 1000 ETB (Ethiopian Birr) (Table 1). About 53.0% of the respondents walk 30 minutes or less to reach the nearest health facility. More than 74.0% of the mothers had at least one ANC visit during their last pregnancy. Similarly, 61.0% of the respondents have received three or more doses of Tetanus Toxoid (TT) vaccine, 56.0% of the mothers gave their last birth in health institutions, and 61.0% had no postnatal checkups. Three hundred forty-four (55.5%) of the respondent had good knowledge about immunization (Table 2).

### 3.2. Immunization Status

Of the total included children (N=620), 77.4% (480/620) of them were fully immunized, 15.5% (96/620) were partially immunized, and the rest 7.1% (44/620) had not received any antigen. On the other hand, of the fully immunized children, 87.3% (419/480) had evidence of immunization supported by the card, while vaccination status of 12.7%% (61/480) of the children was determined by mothers to recall. Similarly, 41.7% (40/96) were confirmed as partially immunized by card, while 58.3% (56/96) were based on mothers' recall.

**Figure 2:**
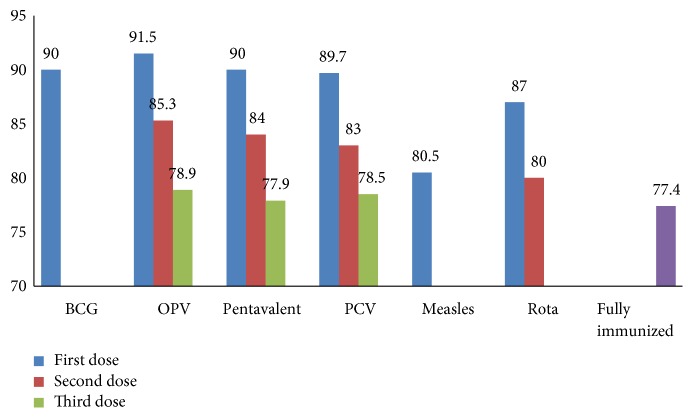
Vaccination coverage among children aged 12-23 months in Sekota Zuria district, Wag-Himra Zone, Northern Ethiopia, 2017.

Overall, 91.5% (567/620) of the children received OPV1, 90.0% (558/620) received both BCG and Pentavalent1, 89.7% (556/620) received PCV1, 80.5% received measles (499/620), and 87.0% (539/620) received the first dose of Rotavirus vaccine. Coverage rates declined for subsequent doses as 78.8% of children received OPV3, 77.3% Pentavalent3, 78.5% PCV3, and 80.0% Rota 2 vaccine. Dropout rate: the proportion of children who started certain vaccine but did not complete the next intended vaccine was 13.8% for OPV1 to OPV3, 13.4% for Pentavalent1 to Pentavalent3, and 10.7% for BCG to Measles (Figure 2).

### 3.3. Factors Associated with Full Immunization Status of the Children

On the bivariable analysis, birth order of the child, mothers' educational status, family size, distance to a health facility, mothers' knowledge score, place of delivery, ANC follow-up, and tetanus toxoid immunization were found to be significantly associated with children's full immunization status. However, in the multivariable analysis, mothers' educational status, place of delivery, mothers' knowledge score, distance to a health facility, family size, and ANC follow-up were found to be significantly associated.

Mothers who attained secondary or more level of education were 2.39 times more likely to have fully immunized children compared to illiterate mothers (Adjusted Odds Ratio (AOR)=2.39, 95%CI=1.06, 5.36). Mothers who travel ≤ 30 minutes to reach the nearest vaccination site were 2.65 times more likely to fully immunize their children than mothers who travel beyond one hour (AOR=2.65, 95%CI= 1.61, 4.36). Mothers who attended ANC services for three or more times were 2.75 times more likely to have fully immunized children compared to mothers who never had ANC visits (AOR=2.75, 95%CI=1.52, 5.00). Children born in health institutions had 2.58 times more chance of being fully immunized than children born at home (AOR=2.58, 95%CI=1.66, 3.99). Mothers who had good knowledge about immunization were 3.7 times more likely to have a fully immunized child as compared to those who had poor knowledge (AOR=3.7, 95%CI=2.37, 5.79). Children born in households with a family size of five and more were 38% less likely to be fully immunized compared to children in households with four and fewer family members (AOR=0.62, 95%CI=0.38, 0.99). (Table 3)

## 4. Discussion 

This study attempts to assess full immunization coverage and its associated factors among children aged 12-23 months in Sekota Zuria District. Our findings revealed that 77.4% of the children were fully immunized during the survey. Mothers' educational status, place of delivery, poor mothers' knowledge, long distance to a health facility, big family size, and ANC follow-up were identified as predictors of full immunization coverage.

The full immunization coverage in the district was higher than the national and regional coverage of the 2016 EDHS report and a study in Areka Town that reported 75.4% [14, 22]. The low proportion of fully immunized children in the EDHS report compared to this district might be due to EDHS data being a national level data and thus a high variability of immunization services [9, 13]. However, Sekou Zuria district had low full immunization coverage as compared to the other similar district-level study that reported 83.1% [3]. It is exclusively rural with hard-to-reach areas. The observed coverage was lower than expected WHO targets of at least 90% by 2015 [6].

Decreasing in coverage rates was observed between the subsequent vaccine doses. The dropout rate observed in this study was lower than the National and Regional findings (20%) [14] but higher than the findings in North West and Southern Ethiopia [9, 23], and more importantly, it exceeds the WHO acceptable dropout limits (>10%) [24]. This decline in subsequent vaccines was possibly due to the forgetfulness, negligence, and low level of mothers' knowledge towards immunization.

Mothers' ANC follow-up positively influenced the immunization status of the children especially among those who attended ANC service at least three times compared to mothers who did not attend ANC at all. This finding is consistent with the other research findings from Nigeria [25], Uganda [26], Kenya [27], and Ethiopia [18, 28]. Similarly, immunization coverage was higher for those who delivered at health institution compared to home delivery. This finding is also in line with a study conducted in Kenya [27] and Ethiopia [17, 19, 23]. The higher chance of getting fully immunized children among children born to women who utilized ANC services or who had institutional delivery could mainly be related to their familiarization with the health care systems during their previous visit and health workers advice.

In this study, maternal education was a predictor of childhood immunization status. The role of maternal education as an important predictor of immunization uptake has also been stated by other studies in Zimbabwe [29], Uganda [26], and Ethiopia [18, 23]. This is due to the contribution of education, changes in attitudes, traditions and beliefs, increased autonomy, and decision-making which could directly enhance a health seeking behavior of the mothers. Apart from maternal education, mothers' good knowledge of immunization programs increased the odds of their children being vaccinated in the district. Research evidence from Nigeria [25] and Southern and North Western Ethiopia reached this similar conclusion [17, 23, 28]. This might be due to mothers having a better understanding of about VPDs, immunization schedules, and awareness about a reason for vaccination, which might increase their motivation to immunize their children.

The geographical accessibility of health facilities has been found to motivate immunization uptake. Mothers who travel for less than an hour to reach their nearest health facility were more likely to have fully immunized children than those who travel beyond one hour. Long distance is a demotivating factor to immunize children. This finding agrees with other studies carried out in Sudan [30], Kenya [27], and Eastern and Southern Ethiopia [3, 23].

Our study found that having a large family size was adversely affected full immunization status of the children. This is in line with studies conducted in Kenya [27] and Ethiopia [11]. Having a large family size might affect the mothers' ability to care for the younger children or the mother might not have enough time to travel for immunization.

In many studies, household economic status was a significant predictor of immunization status [13, 29, 31]. It was argued that children born to economically better HHs have more chance of being vaccinated [7, 8]. In the study area, however, indirect costs like transportation payment are not expected to bring significant differences as the district is already rural and road inaccessible. Other studies in Uganda and Ethiopia had similar findings in which economic status was not a significant predictor of full immunization status of children [9, 26].

Despite generating this important evidence, our study had several limitations such as the following. (1) The effects of health system factors including vaccine availability, health care personnel, and logistics which might have an influence on an uptake of immunizations were not assessed. (2) As data about immunization coverage was collected retrospectively, mothers may not remember all events that took place during an immunization, especially where the card was missing. (3) The study did not address mothers' attitude, perception, and opinion towards children immunization. Despite all these limitations, we hoped our finding would be valuable in bringing updated information on status and barriers of immunization coverage in the rural areas of Ethiopia.

## 5. Conclusion 

Our study indicated that the full immunization coverage of the district was higher than the national and regional coverage, but lower than the World Health Organizations target. Mothers' low educational status, long distance to immunization site, poor mothers' knowledge about immunization, living in large family size, not attending ANC, and institutional delivery were detrimental to achieving full immunization coverage. Improving mother's health seeking behavior toward pregnancy follow-up and enhancing mothers' knowledge on child immunization, strengthening outreach services, community engagement, and actively working with local community-based health agents are recommended to increase number of children to be vaccinated.

## Figures and Tables

**Table 1:** Socio-demographic and economic characteristics of mothers and children aged 12-23 months in Sekota Zuria district, Wag-Himra Zone, North East Ethiopia, 2017.

Characteristics		Frequency (N)	Percentage (%)
Sex of the child	Male	303	48.9
	Female	317	51.1
Birth order of the child	1st	115	18.6
	2nd -3rd	269	43.4
	4th-5th	149	24.0
	≥ 6	87	14.0
Mothers' age in years	≤ 24	160	25.8
	25-34	284	45.8
	≥ 35	176	28.4
Maternal education	Illiterate	309	49.8
	Can read and write	67	10.8
	Primary	171	27.6
	Secondary and above	73	11.8
Marital status	Married	544	87.7
	Single	49	7.9
	Divorced	27	4.4
Mothers' occupation	House wife	169	27.3
	Farmer	331	53.4
	Merchant	54	8.7
	Government employee	25	4.0
	Daily laborer	41	6.6
Religion	Orthodox	590	95.2
	Muslim	30	4.8
Family size	≤ 4	251	40.5
	≥ 5	369	59.5

**Table 2:** Maternal characteristics of mothers and children aged 12-23 months in Sekota Zuria district, Wag-Himra Zone, North East Ethiopia, 2017.

Characteristics		Frequency	Percentage
Place of delivery ANC attended	Health institution	345	55.6
	Home	275	44.4
	None	156	25.2
PNC check ups	1-2 times	269	43.4
	≥ 3 times	195	31.4
	None	378	61.0
TT immunization	Once	162	26.1
	≥ 2 times	80	12.9
	None	137	22.1
Average monthly income	1-2 times	104	16.8
	≥ 3 times	379	61.1
	< 500	213	34.4
	500-1000	253	40.8
	> 1000	154	24.8
Distance to health facility (in walk time)	≤ 30 minutes	328	52.9
	31-60 minutes	135	21.8
	> 60 minutes	157	25.3
Knowledge about immunization	Good	344	55.5
	Poor	276	44.5

**Table 3:** Bi-variable and multivariable analysis of factors associated with full immunization status of children aged 12-23 months in Sekota Zuria district, Wag-Himra Zone, Ethiopia, 2017.

Characteristics	Category	Fully immunized		Odds Ratio (95% CI)	
		Yes	No	COR	AOR
Birth order of the child	1st	94	21	2.12(1.11–4.08)	
	2nd -3rd	212	57	1.77(1.03–3.02)	
	4th -5th	115	34	1.61(0.89–2.89)	
	6th & above	59	28	1	
Mothers' age in years	≤ 24	129	31	1.61(0.96–2.68)	
	25-34	224	60	1.44(0.93–2.23)	
	≥ 35	127	49	1	
Maternal education	Illiterate	224	85	1	1
	Can read & write	52	15	1.32(0.70–2.46)	1.46(0.71–2.15)
	Primary	140	31	1.71(1.08–2.72)	1.62(0.96–2.75)
	2^dary^ & above	64	9	2.69(1.29–5.66)	2.39(1.06–5.36)*∗∗*
Marital status	Married	427	117	1	
	Single	35	14	0.68(0.38–1.32)	
	Divorced	18	9	0.55(0.24–1.25)	
Family size	≤ 4	215	36	1	
	≥ 5	265	104	0.43(0.28–0.65)	0.62(0.38–0.99)*∗∗*
Average monthly income	< 500	160	53	1	
	500-1000	193	60	1.07(0.69–1.63)	
	≥ 1000	127	27	1.56(0.93–2.62)	
Distance to health facility (in walk time)	≤ 30 minutes	278	50	2.83(1.81–4.43)	2.65(1.61–4.36)*∗∗*
	31-60 minutes	98	37	1.35(0.82–2.23)	1.82(1.03–3.23)*∗∗*
	> 60 minutes	104	53	1	1
Exposure to media	None	153	45	1	1
	1-2 times a week	166	61	0.80(0.51–1.25)	0.79(0.48–1.32)
	≥ 3 times a week	161	34	1.39(0.85–2.29)	1.12(0.64–1.97)
Knowledge about immunization	Good	175	101	4.51(2.99–6.83)	3.70(2.37–5.79)*∗∗*
	Poor	305	39	1	1
Place of delivery	Health institution	300	45	3.52(2.36–5.25)	2.58(1.66–3.99)*∗∗*
	Home	180	95	1	1
Frequency of ANC attended	None	103	53	1	1
	1-2	205	64	1.65(1.07–2.54)	1.32(0.81–2.15)
	≥ 3	172	23	3.85(2.23–6.65)	2.75(1.52–5.00)*∗∗*
PNC check ups	None	283	95	1	
	Once	129	33	1.31(0.84–2.05)	
	≥ 2	68	12	1.90(0.99–3.67)	

*∗∗* Significantly associated at p-value ≤ 0.05 in the multi-variable analysis

## Data Availability

The datasets used and/or analyzed during the current study are available from the corresponding author on reasonable request.

## Abbreviations

ANC: Antenatal Care Service
VPD: Vaccine Preventable Disease.
